# Impact of QazVac vaccination on clinical manifestations and immune responses in post—COVID syndrome: a cross-sectional study

**DOI:** 10.3389/fmed.2025.1556623

**Published:** 2025-03-26

**Authors:** Akzhan M. Madenbayeva, Saulesh S. Kurmangaliyeva, Saltanat T. Urazayeva, Kairat B. Kurmangaliyev, Yerlan Sh. Bazargaliyev, Khatimya I. Kudabayeva

**Affiliations:** ^1^Department of Internal Diseases No 1, West Kazakhstan Marat Ospanov Medical University, Aktobe, Kazakhstan; ^2^Department of Microbiology, Virology and Immunology, West Kazakhstan Marat Ospanov Medical University, Aktobe, Kazakhstan; ^3^Department of Epidemiology, West Kazakhstan Marat Ospanov Medical University, Aktobe, Kazakhstan

**Keywords:** post-COVID-19 condition, immunoglobulin G, flow cytometry, QazVac vaccine, cellular immune response

## Abstract

**Introduction:**

Post-COVID syndrome, also known as long COVID, has emerged as a major public health concern, affecting a substantial proportion of individuals recovering from SARS-CoV-2 infection. This condition is characterized by persistent symptoms lasting at least 2 months after acute infection, significantly impacting quality of life and increasing healthcare burdens. In Kazakhstan, the recognition of post- COVID syndrome in national clinical protocols highlights the need for effective prevention and management strategies. Vaccination has been suggested as a key intervention to reduce the severity and prevalence of long COVID symptoms, yet data on its effectiveness, particularly for the domestic QazVac vaccine, remain limited. The aim of this study is to investigate the impact of vaccination with the domestic QazVac vaccine on the features of humoral and cellular immunity in patients with post-COVID conditions and to identify the leading clinical variants of the course.

**Methods:**

We analyzed data from 90 vaccinated and 217 non-vaccinated patients, examining sex, age, smoking status, BMI, comorbidities, and clinical manifestations.

**Results:**

There were no significant differences between the groups with regard to sex, age, and smoking status. However, the characteristics of the subjects indicated that vaccination was correlated with a lower prevalence of diabetes mellitus (2.2% vs. 11.1%, *p* = 0.011) and cardiovascular diseases (0.0% vs. 10.1%, *p* = 0.047), and a higher prevalence of hypertension among non-vaccinated subjects. With regard to clinical symptoms, vaccinated individuals presented a significantly decreased frequency of neurological (51.1% vs. 74.2%, *p* = 0.001), gastrointestinal (4.4% vs. 15.2%, *p* = 0.008), respiratory (21.1% vs. 36.4%, *p* = 0.009), rheumatological symptoms (26.7% vs. 38.7%, *p* = 0.044), and kidney symptoms (2.2% vs. 9.7%, *p* = 0.024). In contrast, unvaccinated participants had more memory loss (49.8% vs. 22.2%, *p* < 0.001), depression (31.3% vs. 6.7%, p < 0.001), joint pain (33.2% vs. 14.4%, *p* = 0.001), and other psychopathological symptoms.

**Discussion:**

A sharp decrease in the frequency of neurological, gastrointestinal, respiratory, and rheumatological symptoms was recorded in vaccinated patients, advocating for the protective role of vaccination against long COVID-19 sequelae. These findings highlight the potential for vaccination to mitigate the burden of post-COVID complications across various organ systems.

## 1 Introduction

Post-COVID syndrome, also referred to as long COVID, has emerged as a significant public health challenge in the aftermath of the COVID-19 pandemic. This condition is characterized by symptoms that persist for at least 2 months following acute SARS-CoV-2 infection and cannot be explained by alternative diagnoses. Due to its prevalence and clinical significance, post-COVID syndrome has been officially recognized as a disease and included in the International Classification of Diseases, 10th Revision (ICD-10), under the code U09.9 (1). According to recent studies, the prevalence of post-COVID syndrome varies widely, with estimates ranging from 10 to 30% of those infected, depending on factors such as disease severity, population demographics, and diagnostic criteria (2, 3). Common manifestations include fatigue, dyspnea, cognitive impairment, and cardiovascular and gastrointestinal symptoms, all of which can significantly impair quality of life and increase healthcare burdens (4). These findings underscore the urgent need to understand and mitigate the long-term consequences of COVID-19.

The relevance of post-COVID syndrome is particularly high in Kazakhstan, where a significant portion of the population suffers from prolonged symptoms following the pandemic (5). On September 16, 2021, the Unified Commission for the Quality of Medical Services under the Ministry of Health developed and approved the first clinical protocol for the diagnosis and treatment of post-COVID syndrome in adults, designated as Protocol No. 147. This protocol has undergone periodic revisions, with the latest being Protocol No. 178, issued on January 30, 2023, highlighting the continued significance of post-COVID syndrome in medical practice in Kazakhstan. Additionally, on April 20, 2022, the Ministry of Health issued Order No. 302, which provided detailed instructions on the organization of medical care and rehabilitation for individuals suffering from post-COVID syndrome (1).

As healthcare systems adapt to managing both acute cases of COVID-19 and long-term complications, the burden of post-COVID syndrome continues to grow (6). National strategies aimed at addressing this issue emphasize the need for comprehensive approaches, including prevention through vaccination and long-term monitoring of patients.

Vaccination remains one of the most effective measures in reducing the severity of COVID-19 and preventing long-term complications such as post-COVID syndrome (7). Clinical studies and observations indicate that vaccinated individuals are less likely to develop severe forms of COVID-19, which may subsequently lower the risk of post-COVID syndrome (8). Recent meta-analyses and systematic reviews confirm that COVID-19 vaccines, particularly mRNA and vector-based platforms, significantly reduce the likelihood of developing severe forms of COVID-19, which may, in turn, lower the risk of post-COVID syndrome. Notably, vaccinated individuals who contract COVID-19 are less likely to experience prolonged symptoms associated with post-COVID syndrome, likely due to faster viral clearance and reduced inflammation (9). COVID-19 vaccination, especially with a two-dose regimen, was associated with a lower risk of long COVID compared to no vaccination and one-dose vaccination (OR, 0.64; 95% CI, 0.45–0.92). Among individuals with ongoing long COVID symptoms, 54.4% did not experience symptomatic changes, while 20.3% showed symptomatic improvement after receiving vaccination (10). The effectiveness of different vaccine platforms, including mRNA vaccines like Pfizer’s Comirnaty and vector-based vaccines like Sputnik, has been a key focus of studies, which have highlighted the role of immune responses in mitigating long COVID outcomes (11).

The immunological mechanisms underlying these effects are complex, with studies indicating that vaccines stimulate both humoral and cellular immunity (12). Recent research has explored the differences in immune responses between vaccine types, particularly regarding their ability to induce long-lasting protection and reduce the severity of long COVID symptoms. Understanding how various vaccine platforms affect immune responses—especially T-cell and antibody production—is critical in assessing their role in preventing post-COVID syndrome (13). In Kazakhstan, several vaccines have been registered for COVID-19 prevention, including vector vaccines [Gam-COVID-Vac, Sputnik Light), inactivated vaccines (QazVac, Vero Cell, CoronaVac, Hayat-Vax), and mRNA vaccines (Comirnaty (Pfizer)].

Among them, the domestic vaccine QazCovid-in, or QazVac, holds a special place. Developed by the Research Institute for Biological Safety Problems, QazVac is based on the classical virus inactivation technology. It is considered one of the first vaccines produced within the Commonwealth of Independent States. QazCovid-in has undergone Phase 1 and Phase 2 clinical trials, demonstrating favorable safety and immunogenicity profiles (14). The vaccine induces a neutralizing antibody response and stimulates a Th1-dependent immune response, which is critical for long-term protection and minimizing the risk of disease development.

Studies confirm that QazVac induces neutralizing antibodies against the SARS-CoV-2 spike protein, a key component for viral entry into human cells (15). However, T-cell immunity was not assessed in this study, leaving a gap in understanding the full spectrum of the vaccine’s immunogenicity and its long-term effectiveness against SARS-CoV-2.

Despite QazVac showing promising results in preventing acute forms of COVID-19, significant gaps remain in understanding its long-term effects, particularly regarding its impact on post-COVID syndrome and the durability of immune responses. Current data on QazVac’s ability to prevent or mitigate post-COVID syndrome are limited, and none of the large-scale studies have evaluated how the vaccine modulates long-term immunity in the population of Kazakhstan. This represents a critical area for further research.

In contrast, other widely used vaccine platforms, such as mRNA vaccines (e.g., Comirnaty (Pfizer)) and vector-based vaccines (e.g., AstraZeneca), have demonstrated distinct immune response profiles. mRNA vaccines have been associated with strong humoral immune responses, rapidly inducing neutralizing antibodies against SARS-CoV-2. These vaccines also tend to provoke a robust T-cell response, which plays a crucial role in long-term immunity and controlling viral replication (16). Conversely, vector-based vaccines, such as AstraZeneca, generate immune responses through the use of a viral vector to deliver genetic material encoding the spike protein. While effective, the immune response induced by vector-based vaccines may differ in terms of magnitude and durability compared to mRNA vaccines. Studies have suggested that mRNA vaccines may offer superior protection against variants of concern and provide stronger long-term immunity, while vector-based vaccines tend to induce more limited immune activation over time (17).

To address these gaps, our study aims to evaluate the impact of QazVac on long-term humoral and cellular immune responses in patients with post-COVID syndrome. We sought to identify the predominant clinical variants of post-COVID syndrome in accordance with the clinical protocol for the diagnosis and treatment of post-COVID syndrome approved in the Republic of Kazakhstan and to assess how QazVac vaccination influences disease progression and symptom severity. By focusing on immune response profiles, clinical manifestations, and vaccination status, this study will provide insights into optimizing strategies for the prevention and treatment of post-COVID syndrome, ultimately enhancing Kazakhstan’s public health response to the long-term effects of the pandemic. The aim of the study is to investigate the impact of vaccination with the domestic QazVac vaccine on the features of humoral and cellular immunity in patients with post-COVID conditions and to identify the leading clinical variants of the course.

## 2 Materials and methods

### 2.1 Study design and participants

This cross-sectional, single-stage, descriptive study was conducted in the Aktobe region of Kazakhstan from November 2022 to January 2024. The study included 307 patients with post-COVID-19 condition, who were divided into two groups based on their vaccination status:

Group I: 90 patients with post-COVID-19 condition, vaccinated with QazVac;

Group II: 217 patients with post-COVID-19 condition, unvaccinated.

Patient recruitment took place at multiple healthcare institutions, including the Family Medicine Clinic of Marat Ospanov West Kazakhstan Medical University, the Multi-Profile Regional Hospital, the Aktobe Medical Center, and City Polyclinic No. 6. Enrollment was facilitated during consultations with various specialists, including infectious disease physicians, general practitioners, and pulmonologists. Individual registration cards (IRCs) were developed and approved for data collection. These IRCs were completed for all patients and included the following sections: personal details, main and additional complaints, medical and life history, physical examination findings, results of instrumental methods (e.g., CT, X-ray, ultrasound), and data from ELISA, PCR, and immunograms.

The diagnosis of post-COVID-19 condition was established in accordance with the clinical protocol for the diagnosis and treatment of post-COVID syndrome in adults. The diagnosis of COVID-19 was confirmed based on positive PCR results in the patient’s medical history, as well as a positive test for antibodies to SARS-CoV-2. The average duration of the COVID-19 illness was 14.5 ± 8.7 days. All participants in the study were thoroughly informed about the research objectives and provided written informed consent to participate in the study.

### 2.2 Ethical considerations

The study adhered to the World Medical Association’s Declaration of Helsinki on ethical principles for medical research involving human subjects. Written informed consent was obtained from all participants before enrollment. The study protocol was approved by the Local Bioethics Committee of Marat Ospanov West Kazakhstan Medical University (Approval No. 7–07, dated September 29, 2022). To ensure participant confidentiality, all personal and medical data were anonymized and securely stored. Only authorized study personnel had access to the data, and all results were reported in aggregate form to maintain privacy.

### 2.3 Inclusion and exclusion criteria

#### 2.3.1 Inclusion criteria

Adults aged 18 to 75 years with a diagnosis of “post-COVID-19 condition” (long COVID syndrome), including both unvaccinated individuals and those vaccinated with the QazVac vaccine. Participation in the study required the expressed willingness of the subject, confirmed by the provision of written informed consent.

#### 2.3.2 Exclusion criteria

Individuals under 18 or over 75 years of age.

Pregnant or breastfeeding women.

Patients with severe comorbidities, including malignancies, autoimmune disorders, or immunodeficiencies.

### 2.4 Data collection and clinical protocol

To study immunological parameters, blood samples were collected approximately 1 year after vaccination, with a single collection performed for all groups. After the diagnosis was established, and upon completing the individual registration card (IRC) and signing the informed consent form, all patients were provided with a referral for a blood test. Patients were instructed on the preparation for the blood test during their visit to the doctor. Blood samples were collected in the procedural room of *In Vivo* Laboratory LLP by an experienced nurse. A total of 10 mL of blood was drawn from the antecubital vein using a disposable needle and distributed into vacuum tubes: one with a yellow cap containing a separating gel and another with a purple cap containing EDTA.

### 2.5 Serological analysis

For immunoenzymatic analysis, a reagent kit from Vector-Best LLC (Russian Federation) was used, designed for the quantitative detection of SARS-CoV-2 immunoglobulin G (IgG), specifically the ELISA-BEST SARS-CoV-2-IgG kit. The quantitative assessment of antibodies to SARS-CoV-2 infection is based on the First International Standard from the World Health Organization (WHO) (code NIBSC: 20/136) and is measured in international units (BAU/mL).

According to the manufacturer’s instructions, the diagnostic sensitivity for detecting IgG to SARS-CoV-2 is 100% (range: 95.7–100, 95% confidence interval), and the diagnostic specificity is 100% (range: 98.5–100, 95% confidence interval). The informational letter accompanying the SARS-CoV-2-IgG quantitative ELISA-BEST reagent kit (No. RZN 2021/14458) specifies that virus-neutralizing activity with a neutralization titer of 1/160 or higher is observed in all samples with specific IgG concentrations of 150 BAU/mL and above (ELISA titer ≥1/600; 95% CI: 83.16–100%). In contrast, only 50% of samples with specific IgG concentrations of 80–149 BAU/mL exhibited virus-neutralizing activity (ELISA titer ≥1/400–1/800; 95% CI: 32.43–67.57%). Specific IgG levels below 10 BAU/mL were considered a negative result for the quantitative analysis.

Blood samples, collected in vacutainers, were labeled with identification numbers, including the participant’s ID and IRC number. The venous blood was left to stand in the tube at room temperature for 30 min until a clot fully formed. After clot retraction, the samples were centrifuged at 3000 rpm for 10 min. The serum was inspected to ensure it was not hemolyzed. Samples were stored and transported at a temperature of 2–8°C for up to 72 h. The first blood sample, collected in a vacutainer with a yellow cap and separating gel, was used to determine the level of IgG antibodies to the SARS-CoV-2 spike protein by ELISA. The results of the quantitative content of anti-SARS-CoV-2 IgG in serum are presented in BAU/mL (binding antibody units/mL), with a reference value of 7.1 BAU/mL. A result >7.1 BAU/mL was considered positive, while a result <7.1 BAU/mL was considered negative.

### 2.6 Flow cytometry for cellular immune analysis

Cellular immune indicators were studied using flow cytometry. Cellular immune parameters were analyzed at the Medical Center of Marat Ospanov West Kazakhstan Medical University. The analysis was performed using a Beckman Coulter Navios flow cytometer (United States) equipped with licensed Kaluza software (version 2.1). This method allows for the qualitative and quantitative measurement of biological and physical properties of immune system cells. Flow cytometry is characterized by high sensitivity, informativeness, and productivity. To ensure standardization, the flow cytometry process was validated, and quality assurance and quality control procedures were implemented. The validation process included equipment certification, analytical method validation, and operator qualification.

Sample preparation: To remove erythrocytes, washing-free technologies were employed using the following lysis solutions: VersaLyse and ImmunoPrep (Beckman Coulter, United States). The intensity of antigen expression was assessed based on the mean fluorescence intensity (MFI, in arbitrary units) using the Beckman Coulter Navios flow cytometer (USA).

**Figure 1:**
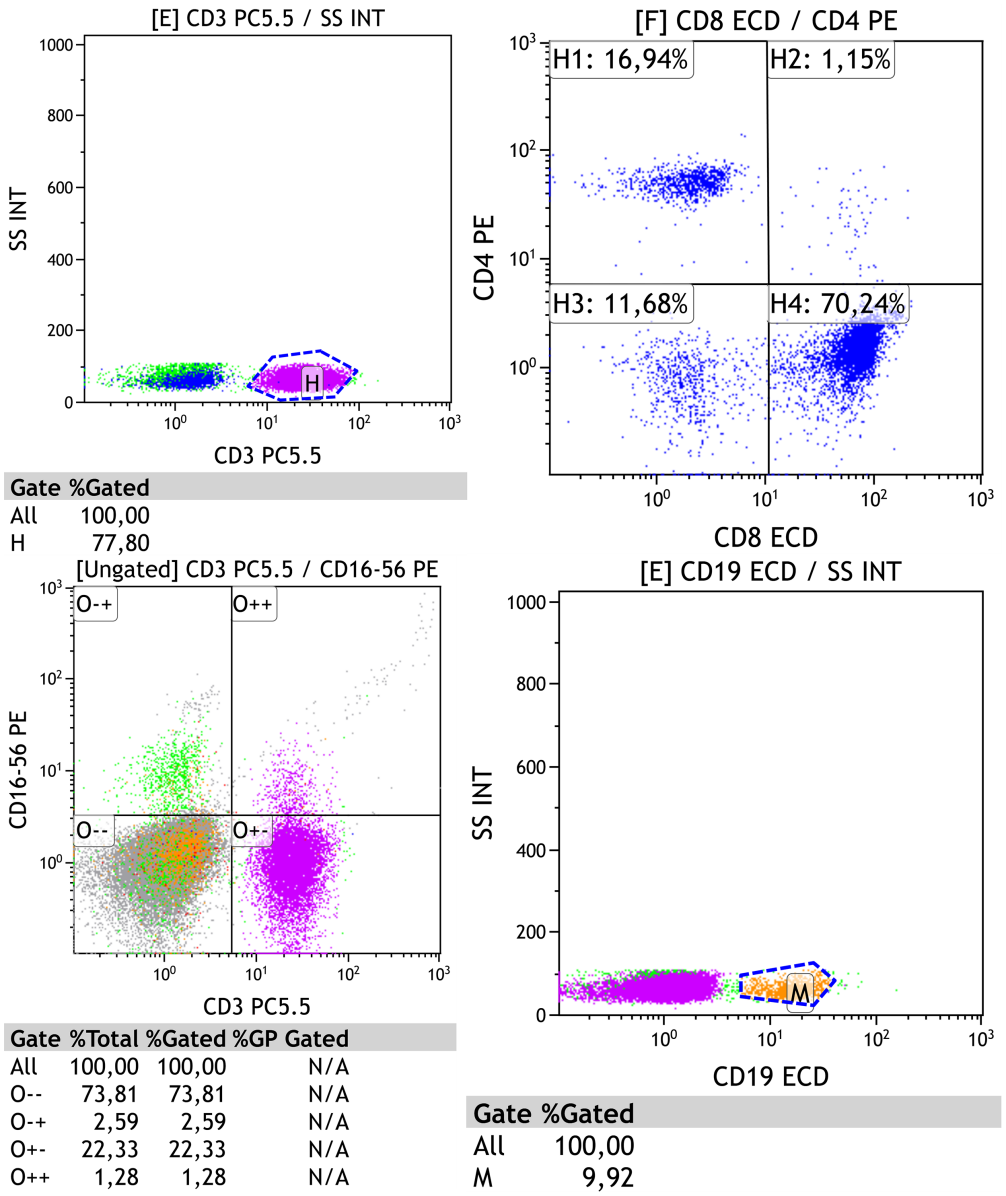
Gating of the main lymphocyte populations: CD3, CD4, CD8, CD16/56, and CD19.

After sample collection, lymphocyte population gating was performed on the flow cytometer based on light scatter parameters. The primary markers used for analysis included the following antigens: CD3, CD4, CD8, CD16/56, and CD19 (Figure 1).

Flow cytometry data analysis involves displaying data from files as a list or gating the population on a two-dimensional graph (e.g., a histogram with one parameter, a two-parameter dot plot, or a three-dimensional plot) and measuring the observed distributions within this graph.

The subpopulation composition of lymphocytes was studied using the following differentiation markers: CD3+, CD3 + CD4+, CD3 + CD8+, CD19+, CD3-CD16 + CD56+, CD3 + CD16 + CD56+, and CD3 + HLA-DR+. The lymphocyte gate, which excludes other blood cells from the analysis, was identified using anti-CD45 monoclonal antibodies (mAb) labeled with PerCP. The analysis was performed using the Beckman Coulter Navios flow cytometer (United States).

### 2.7 Quality assurance

All flow cytometry procedures were conducted by a qualified laboratory diagnostic specialist trained in Flow Cytometry (Certificate No. 0026283, issued by the N.N. Alexandrov Republican Scientific and Practical Center of Oncology and Medical Radiology, Belarus, 2021). Equipment calibration and validation were performed to ensure the accuracy and reproducibility of results.

### 2.8 Statistical analysis

Flow cytometry data were analyzed using SPSS software (version 22). Descriptive statistics were used to summarize demographic and clinical characteristics, with results reported as mean ± standard deviation (SD). For group comparisons, inferential statistical methods, including the independent t-test and Chi-square test, were applied as appropriate. Specifically, the independent t-test was used for comparing continuous variables, while the Chi-square test was employed for categorical data. To account for potential confounding effects of demographic variables (e.g., age, sex, and comorbidities), subgroup analyses and stratified comparisons were conducted. Multivariable regression models were applied to further control for these factors when assessing the association between vaccination status and immune/clinical outcomes.

The sample size was determined based on an *a priori* power analysis to ensure the study had an 80% power (1-*β* = 0.80) to detect a clinically significant difference at a 5% significance level (*α* = 0.05). The power analysis was based on the effect sizes observed in prior studies assessing immune profiles in post-COVID-19 patients and the expected variability within the target population. The calculated sample size was deemed sufficient to detect meaningful differences between the groups, ensuring the robustness and reliability of the study’s findings. A *p*-value of <0.05 was considered statistically significant for all tests. GraphPad Prism software (version 9.5.1, United States) was also used for data visualization.

## 3 Results

### 3.1 Demographic characteristics

In this study, we analyzed the demographic and clinical features of vaccinated (n = 90) and non-vaccinated (*n* = 217) patients with long COVID-19. There was no significant difference in the distribution of sex between the two groups (*p* = 0.635). Among vaccinated patients, 77.8% were female, compared to 80.2% in the non-vaccinated group. Male patients accounted for 22.2 and 19.8% in the vaccinated and non-vaccinated groups, respectively.

Age distribution was also similar between the groups (*p* = 0.081). In vaccinated patients, 32.22% were aged 18–40 years, 47.78% were aged 41–60 years, and 20% were over 60 years. In the non-vaccinated group, these percentages were 31.8, 41.5, and 26.7%, respectively.

#### 3.1.1 Smoking status

Smoking prevalence did not significantly differ between the groups (*p* = 0.107). Among vaccinated patients, 7.8% were smokers, while 92.2% were non-smokers. In the non-vaccinated group, 4.1% were smokers and 95.9% were non-smokers.

#### 3.1.2 Body mass index

BMI categories were similarly distributed between the groups (*p* = 0.942). In vaccinated patients, 2.22% had a BMI < 18.5, 43.34% were in the normal range (18.5–24.9), 40% were overweight (25.0–29.9), and 14.44% were obese (BMI ≥ 30). In the non-vaccinated group, these percentages were 0.92, 45.62, 38.25, and 15.21%, respectively.

#### 3.1.3 Comorbidities

The prevalence of comorbidities varied between the groups. Hypertension was observed in 24.4% of vaccinated patients and 31.8% of non-vaccinated patients, but this difference was not statistically significant (*p* = 0.199). Diabetes mellitus was significantly less common in vaccinated patients (2.2%) compared to non-vaccinated patients (11.1%; *p* = 0.011). Bronchial asthma was reported in 3.3% of vaccinated patients versus 3.7% of non-vaccinated patients (*p* = 0.110). However, cardiovascular diseases were significantly less prevalent among vaccinated patients (0.0%) than in non-vaccinated patients (10.1%; *p* = 0.047). Chronic lung diseases and autoimmune diseases showed no significant differences between the groups (*p* = 0.199 and *p* = 0.449, respectively).

These findings suggest that vaccination status is associated with a lower prevalence of certain comorbidities, particularly diabetes mellitus and cardiovascular diseases, in patients with long COVID-19 (Table 1).

**Table 1:** Comparison of demographic, lifestyle, and clinical characteristics between patients with long COVID-19.

Parameters	Vaccinated patients with long COVID-19 (*n* = 90), *n* (%)	Non-vaccinated patients with long COVID-19 (*n* = 217), *n* (%)	*p*- value
Sex			0.635
Female	70 (77.8%)	174 (80.2%)	
Male	20 (22.2%)	43 (19.8%)	
Age			0.081
18–40 years of age	29 (32.22%)	69 (31.8%)	
41–60 years of age	43 (47.78%)	90 (41.5%)	
> 60 years of age	18 (20%)	58 (26,7%)	
Smoking			0.107
Smokers	7 (7.8%)	9 (4.1%)	
Non-smokers	83 (92.2%)	208 (95.9%)	
Body mass index (BMI)			0.942
<18.5	2 (2.22%)	2 (0.92%)	
18.5–24.9	39 (43.34%)	99 (45.62%)	
25.0–29.9	36 (40.0%)	83 (38.25%)	
>30.0	13 (14.44%)	33 (15.21%)	
Comorbidity			
Hypertension	22 (24.4%)	69 (31.8%)	0.199
Diabetes mellitus	2 (2.2%)	24 (11.1%)	0.011
Cardiovascular diseases	3 (3.3%)	22 (10.1%)	0.047
Bronchial Asthma	0 (0.0%)	8 (3.7%)	0.110
Chronic lung diseases	6 (6.7%)	25 (11.5%)	0.199

### 3.2 Vaccination status affects clinical manifestations of Long COVID-19

There is substantial evidence indicating that the clinical presentation of post-COVID-19 is highly diverse and often challenging to interpret (18). In this study, the identification of symptoms across various clinical forms was guided by the Clinical Protocol for Post-COVID-19 Condition adopted in the Republic of Kazakhstan. All patients demonstrated a wide variability in post-COVID-19 manifestations. Individuals with long COVID-19 were categorized based on their symptoms according to vaccination status and the specific clinical variant observed.

**Figure 2:**
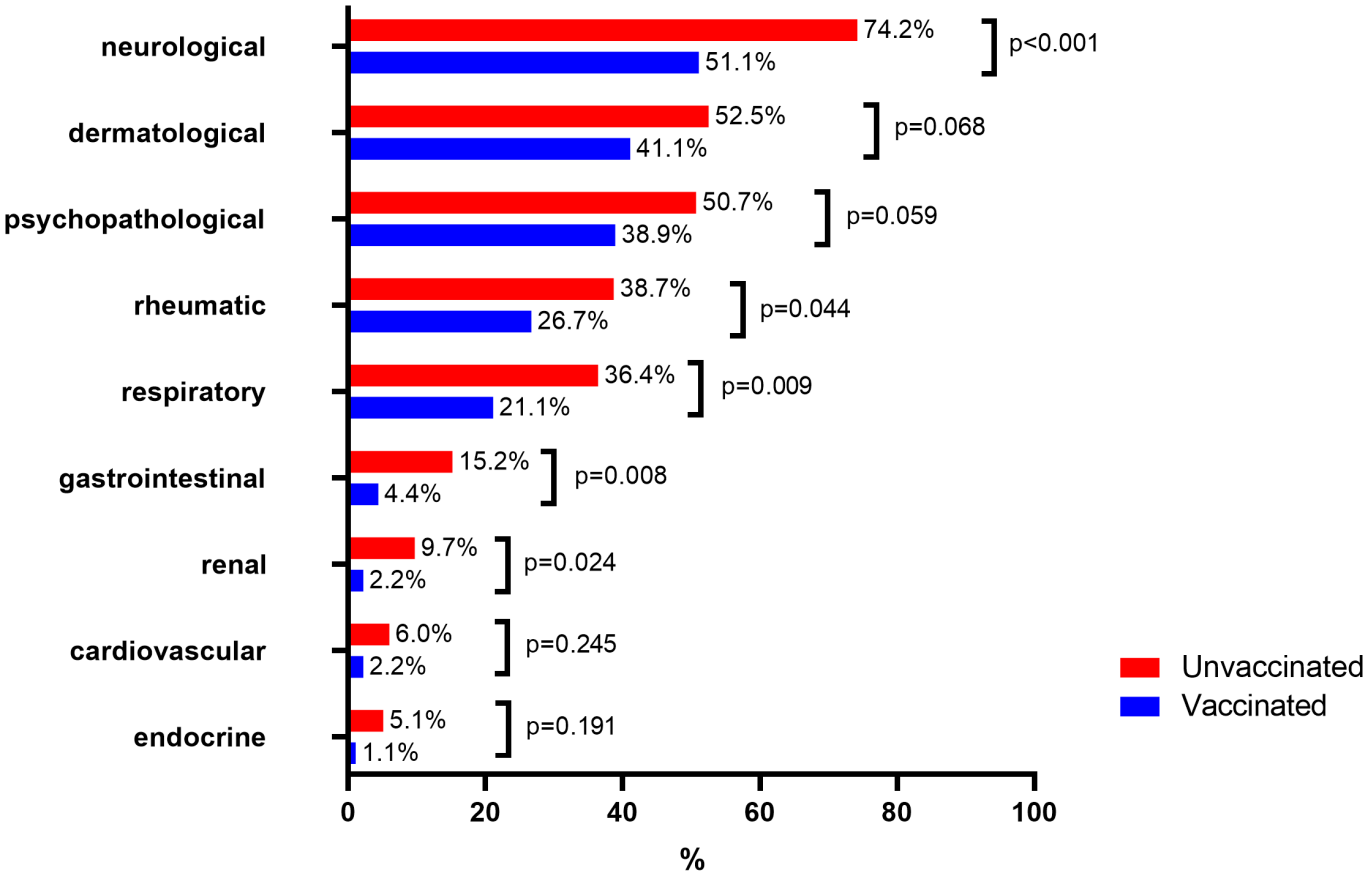
Prevalence of clinical variants in vaccinated and unvaccinated patients with long COVID-19.

Importantly, long COVID-19 frequently involved a broad range of symptoms impacting multiple systems simultaneously (19). However, a detailed analysis conducted in patients with long COVID-19 revealed key clinical variants, including neurological, dermatological, psychopathological, rheumatological, and respiratory forms. Figure 2 provides an overview of the clinical variants observed in post-COVID-19 patients. The data reveal significant differences across various organ systems, with unvaccinated individuals consistently exhibiting higher rates of involvement compared to their vaccinated counterparts.

Neurological manifestations were the most common, observed in 74.2% of unvaccinated individuals compared to 51.1% of vaccinated individuals (*p* = 0.001). This significant reduction highlights the potential protective effect of vaccination against neurological sequelae. A notable difference was seen, with 15.2% of unvaccinated individuals reporting gastrointestinal symptoms compared to 4.4% of vaccinated individuals (*p* = 0.008). Respiratory issues were reported in 36.4% of unvaccinated individuals versus 21.1% of vaccinated individuals (*p* = 0.009). Rheumatic symptoms were present in 38.7% of unvaccinated individuals compared to 26.7% of vaccinated individuals (*p* = 0.044). Renal manifestations were observed in 9.7% of unvaccinated individuals and 2.2% of vaccinated individuals (*p* = 0.024). Other clinical manifestations, including endocrine symptoms (5.1% unvaccinated vs. 1.1% vaccinated, *p* = 0.191), cardiovascular symptoms (6% unvaccinated vs. 2.2% vaccinated, *p* = 0.245), psychopathological symptoms (50.7% unvaccinated vs. 38.9% vaccinated, *p* = 0.059), and dermatological symptoms (52.5% unvaccinated vs. 41.1% vaccinated, *p* = 0.068), showed no statistically significant differences between the groups.

These findings indicate that vaccination is associated with a statistically significant reduction in the prevalence of several clinical symptoms, particularly neurological, gastrointestinal, respiratory, rheumatic, and renal conditions. This underscores the potential role of vaccination in alleviating the burden of post-COVID-19 complications.

**Figure 3:**
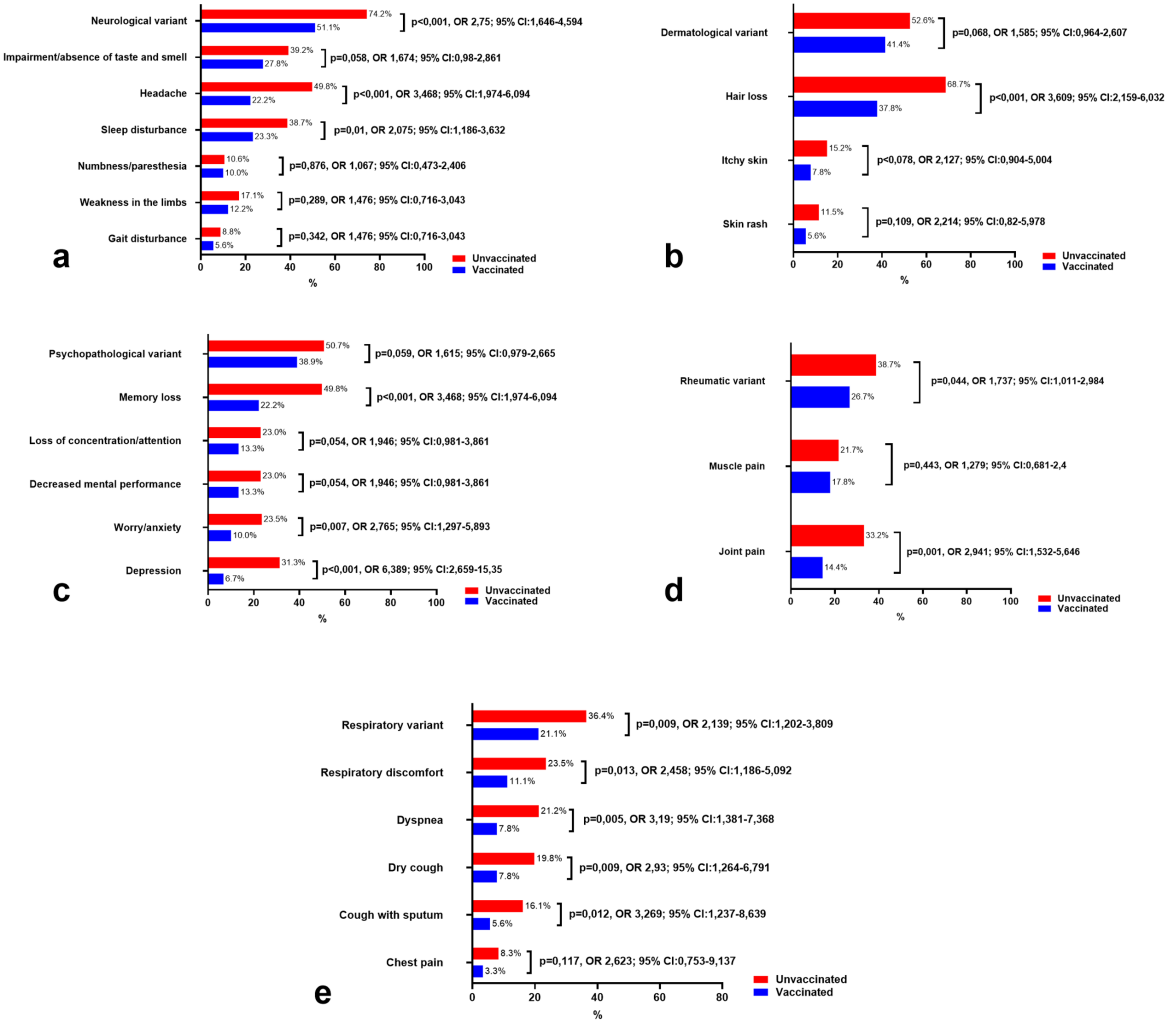
Comparison of the prevalence of leading clinical variants and symptoms of long COVID-19 in vaccinated and unvaccinated patients. **(a)** Symptoms of neurological variant, **(b)** Symptoms of dermatological variant, **(c)** Symptoms of psychopathological variant, **(d)** Symptoms of Rheumatic variant, and **(e)** Symptoms of respiratory variant.

Notably, as illustrated in Figure 3a, the frequency of the neurological variant in vaccinated and unvaccinated individuals differed significantly (*p* < 0.001), with the likelihood of developing neurological symptoms being 2.75 times higher in the unvaccinated group (95% CI: 1.646–4.594). Headache, sleep disturbances, and taste and smell impairment were among the most frequently reported symptoms in unvaccinated individuals, with headaches being significantly more prevalent (*p* < 0.001, OR: 3.468, 95% CI: 1.974–6.094). Symptoms such as numbness, paresthesia, and gait disturbances, indicative of peripheral nervous system damage, were less common in both groups and did not show statistically significant differences.

Interestingly, unvaccinated patients consistently reported higher frequencies of neurological symptoms, even 12 months after the onset of SARS-CoV-2 infection, compared to vaccinated individuals. This trend underscores the potential protective effect of vaccination in reducing the long-term burden of neurological manifestations associated with COVID-19.

Figure 3b summarizes the dermatological symptoms commonly associated with long COVID-19 in both vaccinated and unvaccinated individuals. Hair loss was reported as the most frequent symptom, affecting 37.8% of vaccinated individuals and 68.7% of unvaccinated individuals, with a statistically significant difference between the two groups (*p* < 0.001). Additionally, itchy skin was reported in 7.8% of vaccinated individuals compared to 15.2% of unvaccinated individuals, showing a trend toward increased prevalence in the unvaccinated group, although the difference did not reach statistical significance (*p* = 0.078). Similarly, skin rash was reported in 5.6% of vaccinated individuals and 11.5% of unvaccinated individuals, with no significant difference observed (*p* = 0.109).

Comparing the odds ratios (OR) of dermatological symptoms between the vaccinated and unvaccinated groups, hair loss was found to be significantly more common in unvaccinated individuals (OR = 3.609, 95% CI: 2.159–6.032), indicating a higher likelihood of this symptom in the unvaccinated cohort. For itchy skin, the OR was 2.127 (95% CI: 0.904–5.004), and for skin rash, the OR was 2.214 (95% CI: 0.82–5.978), both suggesting a higher risk in unvaccinated individuals, though the results for itchy skin and skin rash showed wide confidence intervals and did not reach statistical significance.

Figure 3c illustrates the psychopathological symptoms observed in both vaccinated and unvaccinated individuals with long COVID-19. Notably, memory loss was significantly more prevalent in unvaccinated individuals, affecting 49.8% of unvaccinated patients compared to 22.2% in vaccinated patients (*p* < 0.001). The odds of experiencing memory loss were more than three times higher in the unvaccinated group (OR = 3.468, 95% CI: 1.974–6.094), which aligns with previous research highlighting cognitive impairment as a common symptom in post-COVID conditions (20).

Similarly, the prevalence of worry/anxiety was significantly greater in the unvaccinated group, with 23.5% of unvaccinated individuals reporting these symptoms compared to just 10% of vaccinated individuals (*p* = 0.007). The OR for worry/anxiety was 2.765 (95% CI: 1.297–5.893), indicating that unvaccinated individuals were more likely to experience anxiety-related symptoms. Depression was also more common in unvaccinated patients (31.3% vs. 6.7%), with a strikingly high OR of 6.389 (95% CI: 2.659–15.35), suggesting a markedly increased risk of depression in the unvaccinated group (*p* < 0.001).

For other psychopathological symptoms, such as loss of concentration/attention and decreased mental performance, the prevalence was 13.3% in vaccinated individuals and 23% in unvaccinated individuals for both symptoms, with *p*-values of 0.054, showing a trend toward significance. The OR for both symptoms was 1.946 (95% CI: 0.981–3.861), suggesting a higher risk of these symptoms in unvaccinated individuals, but the wide confidence intervals indicate some uncertainty.

Additionally, the psychopathological variant was reported in 38.9% of vaccinated individuals and 50.7% of unvaccinated individuals, with no statistically significant difference (*p* = 0.059). The OR was 1.615 (95% CI: 0.979–2.665), indicating a possible higher prevalence in unvaccinated individuals, though the result did not meet the threshold for statistical significance.

Figure 3d presents the rheumatic symptoms associated with long COVID-19 in both vaccinated and unvaccinated individuals. Joint pain was significantly more common in unvaccinated individuals, affecting 33.2% of unvaccinated patients compared to 14.4% of vaccinated patients (*p* = 0.001). The odds of experiencing joint pain were nearly three times higher in the unvaccinated group (OR = 2.941, 95% CI: 1.532–5.646), suggesting a significant association between unvaccinated status and the occurrence of joint pain in long COVID-19 patients.

The rheumatic variant was found in 26.7% of vaccinated individuals and 38.7% of unvaccinated individuals, with a statistically significant difference between the groups (*p* = 0.044). The OR for the rheumatic variant was 1.737 (95% CI: 1.011–2.984), indicating that unvaccinated individuals were more likely to experience this variant of symptoms, though the confidence interval suggests a moderate level of uncertainty.

In contrast, muscle pain was reported by 17.8% of vaccinated individuals and 21.7% of unvaccinated individuals, with no statistically significant difference observed (*p* = 0.443). The OR for muscle pain was 1.279 (95% CI: 0.681–2.4), showing a slightly higher likelihood of muscle pain in unvaccinated individuals, but the result did not reach statistical significance.

Figure 3e highlights the respiratory symptoms observed in vaccinated and unvaccinated individuals with long COVID-19. The respiratory variant was significantly more prevalent in unvaccinated individuals, affecting 36.4% of unvaccinated patients compared to 21.1% of vaccinated patients (*p* = 0.009). The odds ratio (OR) for the respiratory variant was 2.139 (95% CI: 1.202–3.809), indicating a more than twofold higher likelihood of experiencing respiratory symptoms in the unvaccinated group.

Among specific respiratory symptoms, respiratory discomfort was reported in 23.5% of unvaccinated individuals compared to 11.1% of vaccinated individuals, with a statistically significant difference (*p* = 0.013) and an OR of 2.458 (95% CI: 1.186–5.092). Dyspnea was also significantly more common in unvaccinated patients, affecting 21.2% of unvaccinated individuals compared to 7.8% of vaccinated individuals (*p* = 0.005). The OR for dyspnea was 3.19 (95% CI: 1.381–7.368), indicating a strong association between unvaccinated status and this symptom.

Dry cough and cough with sputum were both more frequent in unvaccinated individuals. Dry cough was reported in 19.8% of unvaccinated patients compared to 7.8% of vaccinated patients (*p* = 0.009, OR = 2.93, 95% CI: 1.264–6.791). Similarly, cough with sputum was observed in 16.1% of unvaccinated individuals compared to 5.6% of vaccinated individuals (*p* = 0.012, OR = 3.269, 95% CI: 1.237–8.639).

Chest pain was less commonly reported but still more prevalent in the unvaccinated group (8.3%) compared to the vaccinated group (3.3%). While the OR for chest pain was 2.623 (95% CI: 0.753–9.137), the difference was not statistically significant (*p* = 0.117), indicating insufficient evidence to conclude a significant association.

As for the remaining variants (gastrointestinal, renal, cardiac, and endocrine), they were observed significantly less frequently in both vaccinated and unvaccinated individuals, with a consistently higher prevalence among the unvaccinated group. While some symptoms showed noticeable differences, such as decreased appetite in the gastrointestinal variant or sallow skin in the renal variant, the overall incidence of these variants remained low. These findings suggest that these manifestations may represent less common post-COVID-19 conditions, with vaccination potentially contributing to their reduced prevalence.

**Figure 4:**
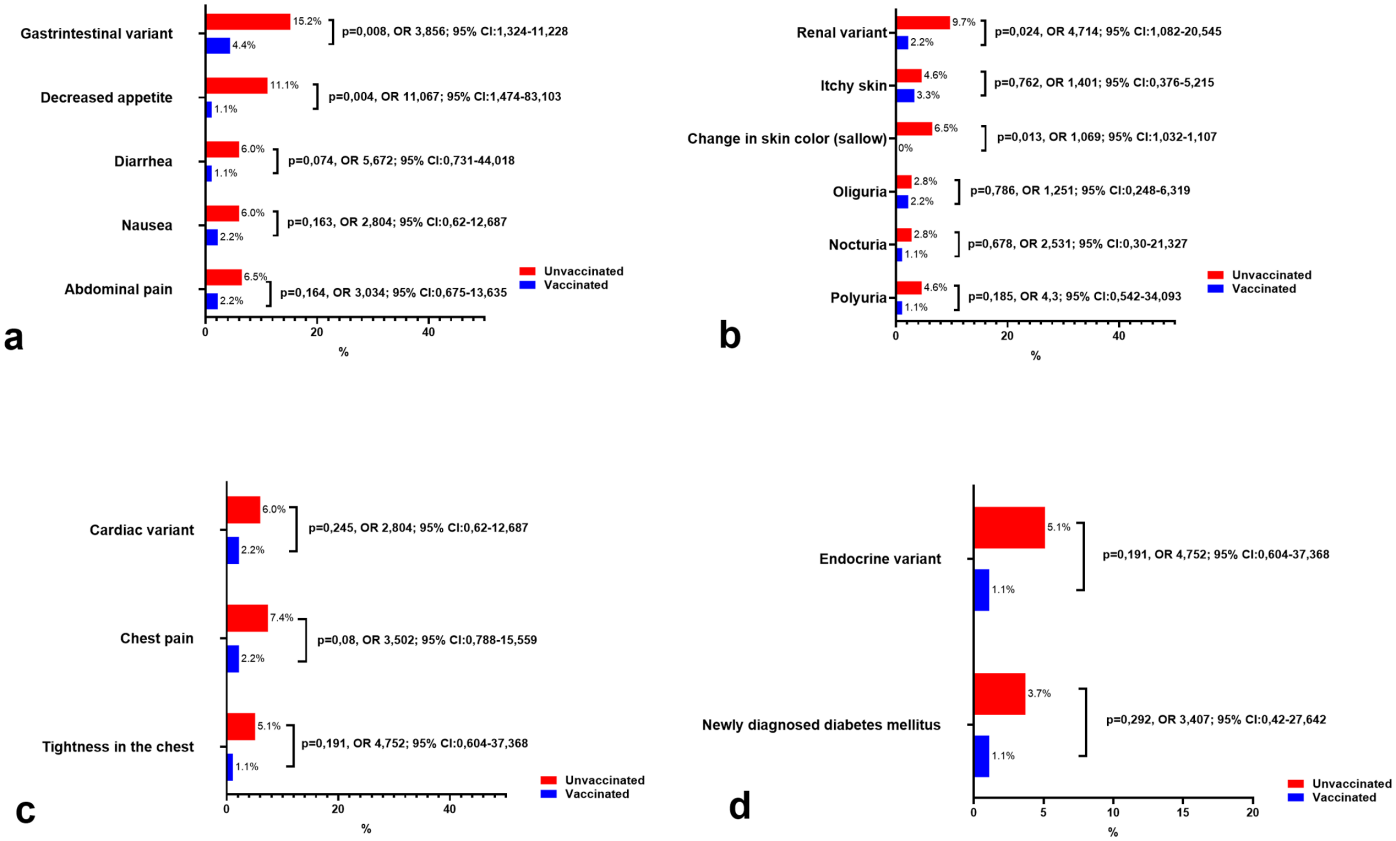
Comparison of the prevalence of remaining clinical variants and symptoms of long COVID-19 syndrome in vaccinated and unvaccinated patients. **(a)** Symptoms of gastrointestinal variant, **(b)** Symptoms of renal variant, **(c)** Symptoms of cardiac variant, and **(d)** Symptoms of endocrine variant.

The gastrointestinal variant (Figure 4a) was more common in unvaccinated individuals (15.2%) compared to vaccinated individuals (4.4%) (*p* = 0.008, OR = 3.856, 95% CI: 1.324–11.228), with decreased appetite being the most pronounced symptom (11.1% vs. 1.1%, *p* = 0.004, OR = 11.067, 95% CI: 1.474–83.103). Other symptoms such as diarrhea, nausea, and abdominal pain were also reported more frequently in unvaccinated patients but remained uncommon overall.

The renal variant (Figure 4b) was similarly rare, affecting 9.7% of unvaccinated individuals and 2.2% of vaccinated individuals (*p* = 0.024, OR = 4.714, 95% CI: 1.082–20.545). Notably, a change in skin color (sallow) was reported exclusively in unvaccinated patients (6.5%, *p* = 0.013), while other renal symptoms, such as oliguria, nocturia, and polyuria, were infrequent and showed no significant differences between groups.

Cardiac symptoms (Figure 4c) were infrequent, with the cardiac variant affecting 6.0% of unvaccinated individuals and 2.2% of vaccinated individuals (*p* = 0.245, OR = 2.804, 95% CI: 0.62–12.687). Chest pain and tightness in the chest were slightly more common in unvaccinated individuals, though these differences did not reach statistical significance.

Finally, the endocrine variant (Figure 4d) was rare, occurring in 5.1% of unvaccinated patients compared to 1.1% of vaccinated individuals (*p* = 0.191, OR = 4.752, 95% CI: 0.604–37.368). Newly diagnosed diabetes mellitus was also more frequent in the unvaccinated group (3.7% vs. 1.1%) but did not demonstrate statistical significance (*p* = 0.292).

### 3.3 Correlation between the age and circulating SARS-CoV-2 S-RBD IgG levels

**Figure 5:**
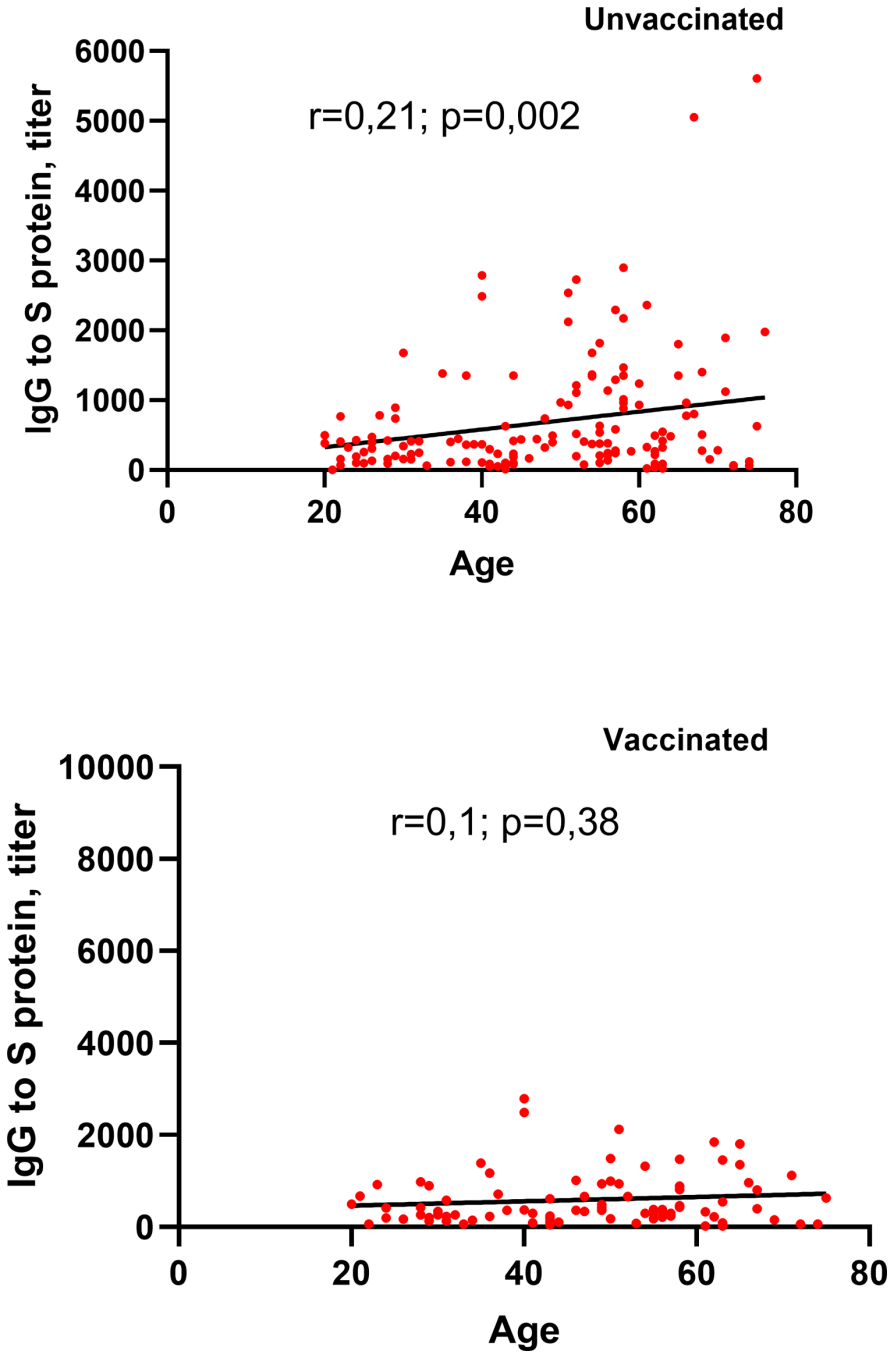
Correlation between age and circulating SARS-CoV-2 S-RBD IgG levels in vaccinated and unvaccinated patients with long COVID-19.

In our study, antibody production was observed across all patients, irrespective of their vaccination status. A correlation analysis was conducted to evaluate the relationship between age and SARS-CoV-2 antibody titers in vaccinated and unvaccinated individuals with post-COVID-19. Among unvaccinated patients, a weak but statistically significant positive correlation between age and antibody titers was detected (r = 0.21, *p* = 0.002; Figure 5). However, in vaccinated individuals, no significant correlation was found between age and antibody titers (r = 0.1, *p* = 0.38).

These findings suggest that in unvaccinated patients, age may play a modest role in influencing antibody levels, while vaccination status appears to mitigate this age-dependent effect on antibody production.

### 3.4 SARS-CoV-2 antibody levels

**Figure 6:**
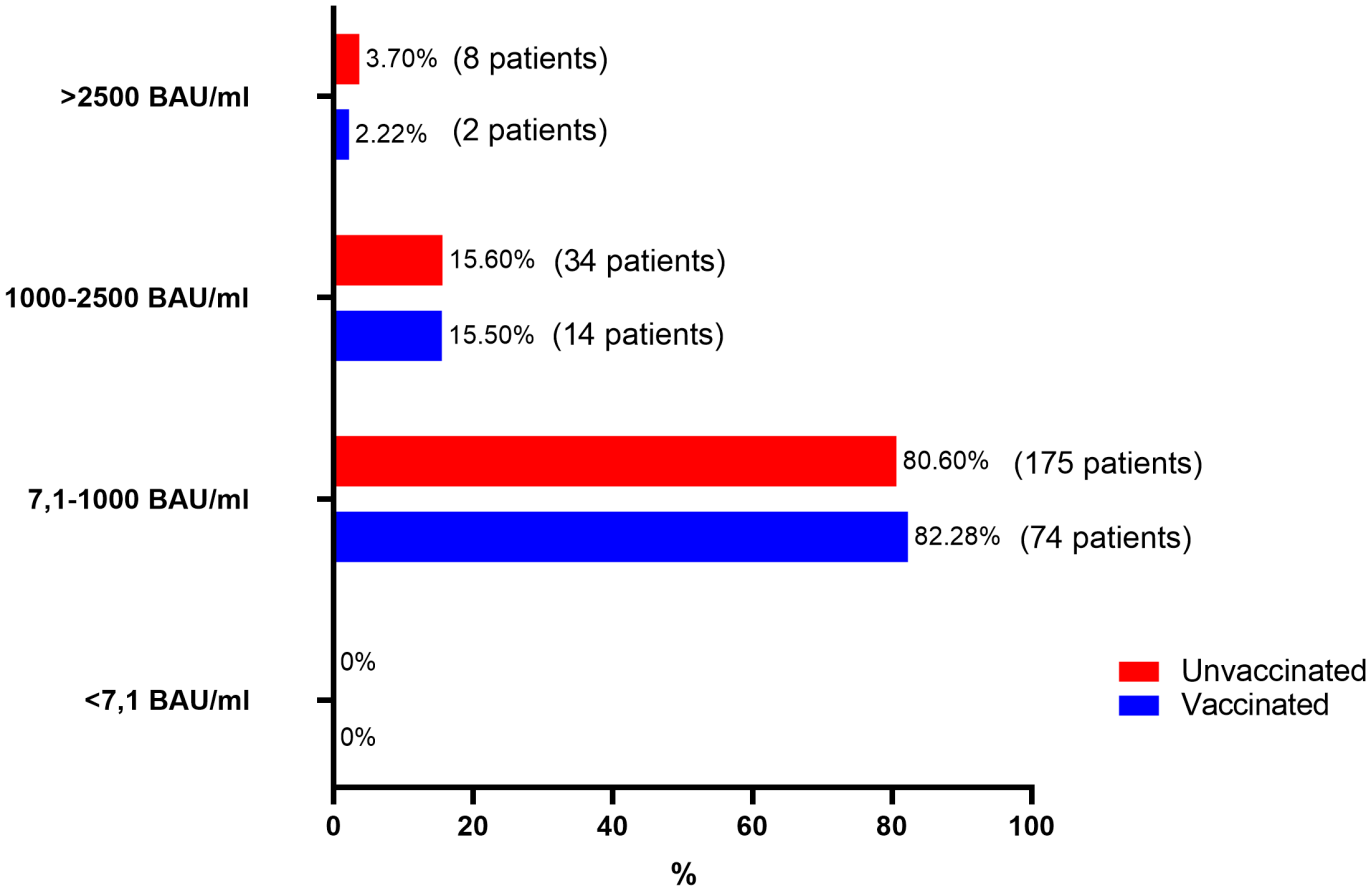
SARS-CoV-2 S-RBD IgG levels in vaccinated and unvaccinated patients with long COVID-19.

As illustrated in Figure 6, circulating SARS-CoV-2 S-RBD IgG levels remained unaffected by vaccination. The analysis of SARS-CoV-2 antibody titers showed no significant differences between vaccinated and unvaccinated groups. None of the participants in either group had antibody levels below 7.1 BAU/mL. The majority of individuals in both groups demonstrated antibody titers within the 7.1–1,000 BAU/mL range, comprising 82.28% of vaccinated individuals and 80.6% of unvaccinated individuals. Moderate antibody levels (1000–2,500 BAU/mL) were observed in 15.5% of vaccinated individuals and 15.6% of unvaccinated individuals, indicating similar distributions. High antibody titers (>2,500 BAU/mL) were rare, reported in 2.22% of vaccinated individuals and 3.7% of unvaccinated individuals. These findings suggest comparable humoral immune responses between the two groups, regardless of vaccination status.

### 3.5 Immune cell populations

**Figure 7:**
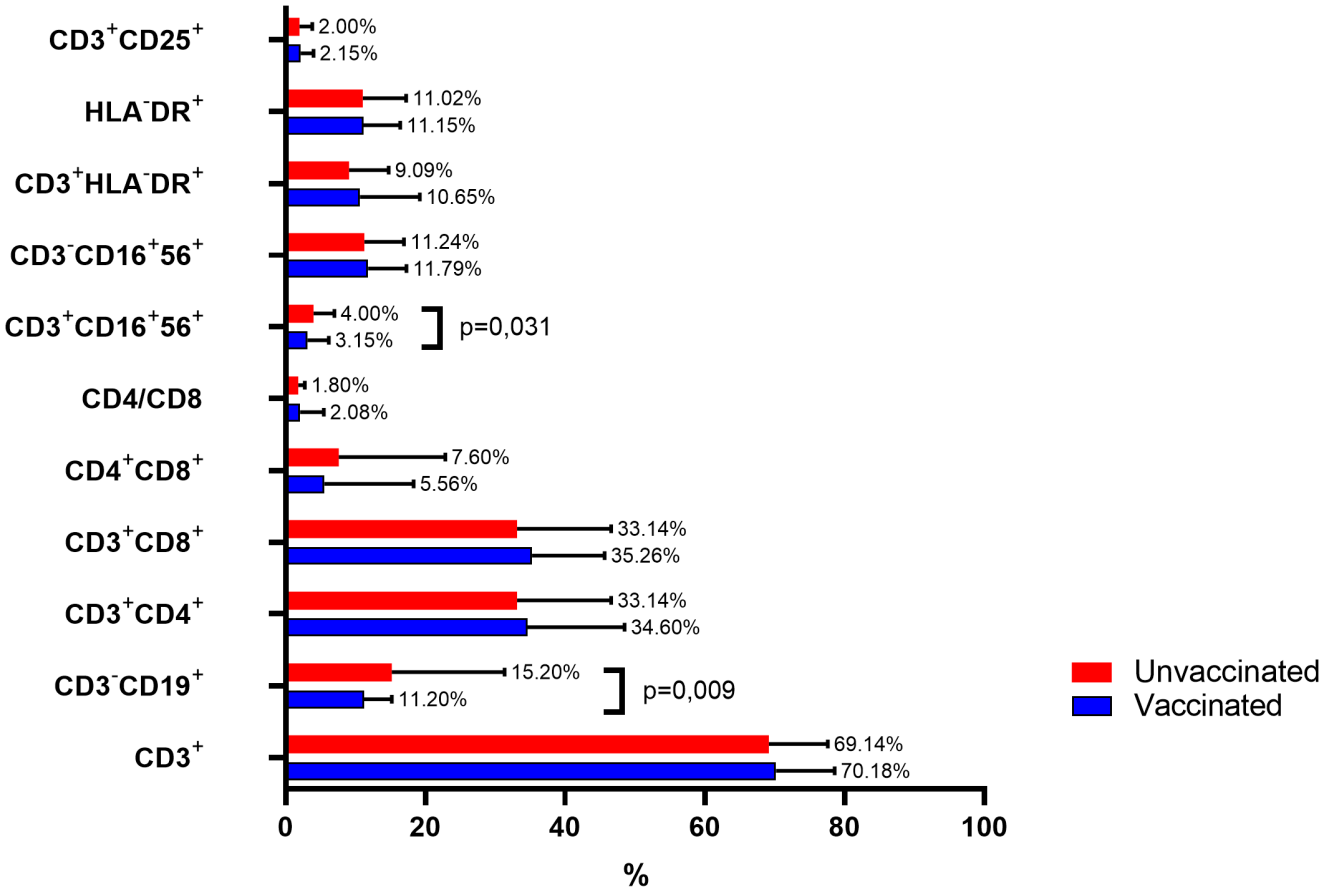
Comparison of immune cell subpopulations in vaccinated and unvaccinated patients with long COVID-19.

Both humoral and cellular immunity are essential components of the immune response following vaccination or natural infection (Figure 7). Cellular immunity, in particular, plays a significant role in antiviral defense mechanisms. An analysis of the immunological status in individuals with long COVID-19 revealed disruptions in certain parameters of cellular immunity. This study aimed to investigate the diversity of cellular subpopulations and explore their potential correlation with protective immunity in post-COVID-19 patients. Specifically, the research focused on examining T-cell and B-cell subpopulations to evaluate their alterations within the framework of hybrid immunity and immunity following natural infection.

Immunophenotyping revealed notable differences in specific lymphocyte subpopulations between vaccinated and unvaccinated patients with post-COVID-19, while most subsets remained comparable.

CD3+ T cells: The percentages were similar between groups, with vaccinated individuals showing 70.18 ± 8.38% and unvaccinated individuals 69.14 ± 8.45%.

CD3-CD19+ B cells: This population was significantly lower in vaccinated patients (11.2 ± 3.98%) compared to unvaccinated individuals (15.2 ± 16.13%, *p* = 0.009).

CD4+ helper T cells: Constituted 34.6 ± 13.9% in vaccinated individuals and 33.14 ± 13.45% in unvaccinated patients.

CD8+ cytotoxic T cells: Observed at comparable levels (35.26 ± 10.37% in vaccinated vs. 33.14 ± 13.45% in unvaccinated).

Double-positive CD4 + CD8+ T cells: More prevalent in unvaccinated individuals (7.6 ± 15.26%) compared to vaccinated individuals (5.56 ± 12.74%), though this difference was not statistically significant.

CD4/CD8 ratio: Slightly higher in vaccinated individuals (2.08 ± 3.37) compared to the unvaccinated group (1.8 ± 0.942).

Natural killer (NK) T cells (CD3 + CD16 + 56+): Significantly reduced in vaccinated individuals (3.15 ± 3.02%) compared to unvaccinated individuals (4.0 ± 3.0%, *p* = 0.031).

CD3-CD16 + 56+ NK cells: No significant differences were observed (11.79 ± 5.5% in vaccinated vs. 11.24 ± 5.67% in unvaccinated).

Activated T cells (CD3 + HLA-DR+): Comparable between groups (vaccinated: 10.65 ± 8.56%; unvaccinated: 9.09 ± 5.67%).

HLA-DR+ expression across all lymphocytes: Similar in both groups (vaccinated: 11.15 ± 5.24%; unvaccinated: 11.02 ± 6.24%).

Regulatory T cells (CD3 + CD25+): Similarly distributed between groups (vaccinated: 2.15 ± 1.85%; unvaccinated: 2.0 ± 1.83%).

These findings highlight significant differences in B cell and NK T cell populations, particularly reductions in CD3-CD19+ B cells and CD3 + CD16 + 56+ T cells among vaccinated individuals, suggesting a potential impact of vaccination on specific immune cell subsets in post-COVID-19 patients.

## 4 Discussion

This study describes the clinical and immunological profiles of individuals with post-COVID-19 conditions, comparing fully vaccinated individuals with QazVac to unvaccinated individuals. The results highlight the potential protective effects of vaccination during post-COVID-19 events and its role in modulating immune cell behavior. Statistical analyses revealed no significant differences in sex, age distribution, smoking status, or BMI between the groups, suggesting a similar baseline demographic profile. However, vaccinated patients had a lower prevalence of certain comorbidities, particularly diabetes mellitus and cardiovascular diseases. These findings align with previous studies indicating that vaccination reduces systemic inflammation and improves health outcomes in at-risk populations (21, 22).

The clinical spectrum of long COVID-19 was broad, with unvaccinated individuals showing significantly higher odds of experiencing neurological, gastrointestinal, respiratory, rheumatic, and renal symptoms. Neurological manifestations were the most predominant in both groups but were more frequent in unvaccinated patients (74.2% vs. 51.1%, *p* = 0.001). These findings are consistent with existing literature reporting that vaccination reduces the risk of neuroinflammatory sequelae, which may stem from altered immune responses and decreased CNS viral persistence (23).

Similarly, gastrointestinal, respiratory, and rheumatic symptoms were also less prevalent among vaccinated individuals. The reduced odds of gastrointestinal and respiratory symptoms in vaccinated patients may indicate enhanced mucosal immunity and a decreased inflammatory response following infection (24, 25). Clinical manifestations included psychopathological symptoms such as memory loss, anxiety, and depression, which were significantly more frequent among the unvaccinated. Memory loss, reported in 49.8% of unvaccinated patients, was over three times more common than in vaccinated individuals. This finding supports studies identifying cognitive dysfunction as a hallmark of long COVID-19, likely exacerbated by chronic inflammation and endothelial dysfunction (26). The lower prevalence of anxiety and depression in vaccinated individuals may reflect not only the biological effects of vaccination on inflammatory pathways linked to mood disorders but also the psychological reassurance associated with vaccination (27).

Hair loss, a common dermatological manifestation, was up to twice as prevalent in unvaccinated individuals (68.7% vs. 37.8%, *p* < 0.001). Previous studies suggest that SARS-CoV-2 may directly affect keratinocytes, leading to hair follicle damage (28). Vaccination may mitigate this by reducing viral load and systemic inflammation (29).

Unvaccinated patients also exhibited significantly higher rates of rheumatic symptoms, particularly joint pain (33.2% vs. 14.4%, *p* = 0.001). The apparent protective effect of vaccination may be attributed to reduced immune dysregulation and autoimmunity, which are key drivers of post-viral rheumatic complaints (30). The study further highlights similar humoral immune responses between vaccinated and unvaccinated individuals, as no significant differences were found in SARS-CoV-2 S-RBD IgG antibody titers. This suggests a robust humoral response in both groups, consistent with previous reports (31).

A notable aspect of this study was the examination of differences in specific immune cell subsets, emphasizing their potential role in disease progression and recovery. T-cell-mediated immunity plays a crucial role in controlling SARS-CoV-2 infection, mitigating disease severity, and potentially reducing the risk of long-term complications (32). Levels of CD3^−^CD19^+^ B cells and CD3^+^CD16^+^56^+^ NK T cells were lower in vaccinated individuals. These reductions may indicate a modulation of both adaptive and innate immune responses following vaccination, potentially reflecting a quicker resolution of inflammation and immune activation (33).

One of the most striking findings was the observed reduction in B cell populations (CD3^−^CD19^+^ B cells) in vaccinated individuals. This could suggest a more efficient immune response post-vaccination, where a lower B cell count reflects adaptive immune system modulation, allowing for faster resolution of acute inflammation (34). A reduction in B cells may also indicate less prolonged immune activation, contributing to lower chronic inflammation and a reduced risk of long COVID symptoms. This supports the idea that vaccination facilitates a controlled and swift immune response, limiting persistent immune activation that could otherwise lead to post-viral inflammation (35).

In contrast, the lower levels of NK T cells (CD3^+^CD16^+^56^+^) observed in vaccinated individuals provide additional insights into immune regulation after vaccination (36). NK T cells are crucial in the innate immune response, playing roles in viral replication control and inflammation modulation. Their reduced numbers in vaccinated individuals may indicate that the immune system has effectively managed the acute phase of infection, leading to less immune dysregulation and a more balanced inflammatory response post-infection (37). This reduction may also correlate with a decreased systemic inflammatory state, which has been implicated in long COVID symptoms such as fatigue and cognitive dysfunction (38).

The significantly higher frequency of double-positive CD4^+^CD8^+^ T cells in unvaccinated individuals could indicate persistent inflammation, a characteristic feature of post-viral syndromes. These cells are typically found in individuals experiencing ongoing immune dysregulation, which may contribute to chronic inflammation and the persistence of long COVID symptoms (39, 40). Their increased prevalence in unvaccinated individuals suggests prolonged immune activation, potentially explaining the higher incidence of persistent symptoms, including neurological, gastrointestinal, and rheumatic manifestations. This aligns with previous studies emphasizing the role of sustained immune activation in long COVID pathophysiology (41, 42).

The higher CD4/CD8 ratio in vaccinated individuals may suggest a more balanced adaptive immune response and a lower risk of chronic inflammation (43). Additionally, this study provides insight into hybrid immunity, which results from the combination of natural infection and vaccination. Both groups demonstrated strong humoral responses, but vaccinated individuals exhibited distinct cellular immune profiles that may enhance protection against severe or long COVID-19. The data suggest that the interaction between decreased B cells and higher NK cells in vaccinated individuals may contribute to more durable immunity.

### 4.1 Limitations of the study

This observational study has several limitations. A major limitation is the potential for recall bias, as it relies on self-reported symptoms and vaccination records. Participants may have inaccurately reported their symptoms or vaccination history, leading to potential data biases. While efforts were made to validate records, the reliance on participant recall could have affected the accuracy of the findings.

The study’s follow-up period was relatively short, limiting the ability to assess the long-term effects of post-COVID syndrome and vaccine effectiveness. A longer follow-up is needed to capture delayed-onset symptoms and changes in immune profiles over time. Future studies with extended follow-up periods could provide a more comprehensive understanding of the prolonged effects of post-COVID syndrome and the durability of vaccine-induced protection.

Additionally, the study was geographically limited to Kazakhstan, which may affect the generalizability of the findings to other regions with different demographic profiles or healthcare systems. The results may not be directly applicable to populations outside Kazakhstan, particularly those receiving different vaccines. Since this study focused specifically on the QazCovid-in vaccine, the findings may not fully represent the effects of other vaccine platforms, such as mRNA vaccines (e.g., Pfizer) or vector-based vaccines (e.g., AstraZeneca), which are widely used in other parts of the world.

To address these limitations, we recommend future studies incorporating prospective cohort designs, more diverse populations, and a broader range of vaccine platforms. Such studies would help clarify the long-term impact of different vaccines on post-COVID syndrome and immune responses across various regions and populations.

## 5 Conclusion

Overall, the data from this study demonstrate the protective effects of the QazVac vaccine in mitigating the severity of post-COVID-19 conditions and modulating the immune response. Vaccinated individuals exhibited lower rates of neurological, gastrointestinal, respiratory, rheumatic, and dermatological symptoms, as well as less severe symptoms compared to unvaccinated individuals. This further supports the theory that vaccination helps reduce inflammation and viral persistence.

While both groups displayed similar humoral immune responses, vaccinated individuals exhibited distinct cellular immune profiles, characterized by lower levels of CD3-CD19+ B cells and CD3 + CD16 + 56+ NK T cells in response to SARS-CoV-2 infection. This suggests that vaccinated individuals mounted a more effective immune response after initial exposure, limiting subsequent viral replication and providing protection against reinfection. In contrast, unvaccinated individuals showed a higher prevalence of double-positive CD4 + CD8+ T cells, indicating persistent immune dysregulation post-infection (Figure 3).

These findings support the concept of hybrid immunity, in which vaccination enhances protection against severe post-COVID-19 conditions. Beyond clinical management, these results have important public health implications, as vaccination appears to be a key strategy in reducing the burden of long COVID. Further research is needed to elucidate the mechanisms underlying these immune changes and to assess the long-term effectiveness of vaccination.

## Data Availability

The original contributions presented in the study are included in the article/supplementary material, further inquiries can be directed to the corresponding author.

## Glossary

Post-COVID Syndrome (Long COVID): A condition characterized by persistent symptoms lasting at least 2 months after an acute SARS-CoV-2 infection, which cannot be explained by other causes.
Immune Response: The body’s defense mechanism against pathogens, involving both innate (immediate) and adaptive (long-term) immunity. Post-COVID syndrome is thought to be influenced by persistent immune activation and dysregulation.
Humoral Immunity: The aspect of immunity mediated by antibodies produced by B cells, which play a critical role in neutralizing SARS-CoV-2 and preventing reinfection.
Cellular Immunity: An immune response involving T cells (CD4+ helper T cells and CD8+ cytotoxic T cells) that help eliminate infected cells and regulate long-term immune memory.
Vaccination: The administration of a vaccine to stimulate an immune response that protects against infectious diseases. COVID-19 vaccines, including mRNA (e.g., Pfizer-BioNTech), vector-based (e.g., AstraZeneca), and inactivated vaccines (e.g., QazVac), have been developed to prevent severe disease and post-COVID complications.
QazVac (QazCovid-in): A domestically developed inactivated COVID-19 vaccine from Kazakhstan, created by the Research Institute for Biological Safety Problems. It is designed to generate neutralizing antibodies and provide long-term immunity against SARS-CoV-2.
mRNA Vaccines: A vaccine technology that uses messenger RNA (mRNA) to instruct cells to produce the SARS-CoV-2 spike protein, stimulating an immune response. Examples include Pfizer-BioNTech’s Comirnaty and Moderna’s Spikevax.
Vector-Based Vaccines: A type of vaccine that uses a viral vector (such as adenovirus) to deliver genetic instructions for producing the spike protein, triggering an immune response. Examples include AstraZeneca and Sputnik V.
Hybrid Immunity: Immunity acquired through a combination of natural infection and vaccination, leading to enhanced protection against COVID-19 and its long-term complications.
Cytokine Storm: A severe immune response characterized by excessive cytokine production, leading to systemic inflammation, organ damage, and complications in COVID-19. Persistent immune activation after infection may contribute to post-COVID syndrome.
CD3+ T Cells: A broad category of T lymphocytes expressing the CD3 marker, essential for T cell receptor (TCR) signaling and immune activation. These cells include both CD4+ helper T cells and CD8+ cytotoxic T cells.
CD4+ Helper T Cells: A subset of T lymphocytes that coordinate immune responses by secreting cytokines and activating other immune cells, including B cells and cytotoxic T cells. They play a critical role in adaptive immunity and immune memory formation.
CD8+ Cytotoxic T Cells: Effector T cells that recognize and eliminate virus-infected or malignant cells through direct cytotoxic mechanisms, such as perforin- and granzyme-mediated apoptosis.
CD4 + CD8+ Double-Positive T Cells: A transitional subset of T lymphocytes that co-express CD4 and CD8 markers, typically observed in the thymus during T cell maturation. Their persistence in peripheral blood may indicate immune dysregulation or activation.
CD3-CD19+ B Cells: A major subset of B lymphocytes responsible for antibody production and antigen presentation. Their frequency can be affected by infections, autoimmune diseases, or vaccination.
CD3 + CD16 + CD56+ Natural Killer (NK) T Cells: A hybrid subset of T cells sharing features of both conventional T cells and NK cells. They play a role in tumor surveillance, antiviral immunity, and immune modulation.
CD3-CD16 + CD56+ Natural Killer (NK) Cells: Innate immune cells with cytotoxic functions, capable of targeting virus-infected and transformed cells without prior antigen sensitization. They contribute to early immune responses and immune regulation.
CD3 + HLA-DR+ Activated T Cells: A subset of T cells expressing HLA-DR, a marker of activation. These cells are typically enriched in inflammatory conditions and may indicate immune system overactivity or chronic immune stimulation.
CD3 + CD25+ Regulatory T Cells (Tregs): A specialized subset of T cells that modulate immune responses and maintain self-tolerance, preventing excessive inflammation and autoimmune reactions.
CD4/CD8 Ratio: A commonly used immunological parameter reflecting immune system balance. A lower ratio is often associated with immune suppression, while a higher ratio may indicate immune activation or dysregulation.
